# Association between primary angle closure glaucoma and uric acid levels in serum and aqueous humor

**DOI:** 10.1016/j.heliyon.2024.e30721

**Published:** 2024-05-03

**Authors:** Wei Liu, Ruru Guo, Fei Gao, Dandan Huang, Xinyi Zhang, Jian Ji, Nomdo M. Jansonius

**Affiliations:** a Tianjin Key Laboratory of Retinal Functions and Diseases, Tianjin Branch of National Clinical Research Center for Ocular Disease, Eye Institute and School of Optometry, Tianjin Medical University Eye Hospital, Tianjin, 300384, China; b Department of Ophthalmology, University of Groningen, University Medical Center Groningen, Groningen, the Netherlands; c Department of Ophthalmology, Taihe Hospital, Hubei University of Medicine, Shiyan, Hubei, China

**Keywords:** Primary angle closure glaucoma, Uric acid, Oxidative stress, Aqueous humor

## Abstract

**Purpose:**

To evaluate abnormalities in serum and aqueous humor uric acid (UA) levels in primary angle closure glaucoma (PACG).

**Methods:**

Patients with PACG and age-similar and gender-similar controls (patients scheduled for cataract extraction) were enrolled prospectively. Serum UA levels were determined by enzymatic colorimetry; aqueous humor UA levels by Enzyme-Linked ImmunoSorbent Assay. A *t*-test was used to compare UA levels between PACG patients and controls, with one-way ANOVA used to compare levels across PACG subgroups with differing disease severity. Comparisons between PACG patients and controls were adjusted for systemic and ocular confounding factors using binary logistic regression.

**Results:**

In all, 131 PACG patients and 112 controls were included. The serum UA level was 266 ± 69 μmol/L in the PACG group and 269 ± 73 μmol/L in the control group (p = 0.71). The aqueous humor UA level was 35.4 ± 8.2 μmol/L in the PACG group and 53.9 ± 18.6 μmol/L in the control group (p < 0.001). This difference remained significant after adjusting for age, gender, systolic blood pressure, diastolic blood pressure, body mass index, axial length, central corneal thickness, anterior chamber depth, lens thickness, white-to-white distance, corneal endothelial cell density, and serum UA level (odds ratio: 0.88, 95 % confidence interval: 0.83–0.93, p < 0.001).

**Conclusion:**

Aqueous humor UA levels differ between PACG patients and controls, but serum UA levels do not. This indicates that local UA plays a role in the pathogenesis of PACG, but systemic UA does not.

## 1 Introduction

Glaucoma—the primary contributor to irreversible blindness worldwide—is characterized by degeneration of the retinal ganglion cells, leading to optic disc cupping and corresponding visual field defects. Based on the status of the anterior chamber angle, glaucoma is commonly divided into open angle glaucoma and angle closure glaucoma. Primary open angle glaucoma (POAG) is the predominant subtype among people of Caucasian, African, and Hispanic people, while primary angle closure glaucoma (PACG) is the prevailing subtype among Asian and Inuit people [1]. Characterized by elevation of the intraocular pressure (IOP), PACG results from the mechanical blockage of the trabecular meshwork (TM) by either appositional or synechial angle closure. It results in a three times higher risk of bilateral visual impairment in comparison with POAG [2]. Although IOP is regarded as the main risk factor and the only modifiable factor in both POAG and PACG, the amount of damage given a certain level and duration of IOP elevation varies widely across patients.

Glaucoma has a complex, multifactorial pathophysiology, which is not fully elucidated. Recently, the role of uric acid (UA) in the pathophysiology of glaucoma has become a topic of investigation. UA can be hypothesized both to be harmful (via inducing oxidative stress and provoking endothelial dysfunction) [3] and to be protective (via its antioxidant properties) [4,5]. Possibly in line with this, the results of various clinical and epidemiological studies on the role of UA in POAG were inconsistent, with evidence of both increased and decreased serum UA levels (for review see Liu et al. [6]). Researchers from Tunis [7] and Greece [8] found that the serum UA levels of POAG patients were higher than that of controls, while lower serum UA levels in POAG patients were found in two other studies, one from China [9] and one from Italy [10]. In PACG, the literature is limited to two studies, both reporting decreased serum UA levels in PACG [11,12]. These two studies are from the same research team and are both clinic based. In a recent meta-analysis including POAG, PACG, and normal-tension glaucoma patients, the authors found that glaucoma patients had a non-significantly higher serum UA level compared to controls [13]. Clearly, further research is needed to elucidate the exact relationship between UA and glaucoma. Moreover, besides the serum UA levels, changes in the local environment (e.g., aqueous humor UA) may also contribute to the onset and progression of glaucoma [14].

In this study, we aimed to further insight into the systemic and local UA levels in glaucoma. For this purpose, we measured both serum and aqueous humor UA levels in a large group of PACG patients and controls.

## 2 Methods

This study was approved by the ethics committee of Tianjin Medical University Eye Hospital (2018KY-02) and adhered to the tenets of the Declaration of Helsinki. PACG patients and controls with cataract who were scheduled for surgery from May 2019 to June 2020 were collected prospectively. Written informed consent was acquired from all participants, after providing a comprehensive description of the study's purpose and potential implications. This study was registered in the Chinese Clinical Trial Registry (No. ChiCTR1800017469) on July 31, 2018.

### 2.1 Patients and controls

Patients with PACG and controls were recruited prospectively. For inclusion, their age had to fall within the range from 18 to 80 years. For the patients with PACG, we consecutively included those who were scheduled for trabeculectomy or trabeculectomy combined with cataract surgery in the Tianjin Medical University Eye Hospital and who fulfilled the exclusion and inclusion criteria. For the control subjects, we consecutively recruited cataract patients who were scheduled for cataract surgery. The diagnosis of PACG was based on the International Society of Geographic and Epidemiologic Ophthalmology criteria: eyes with narrow angles (a minimum of 180 degrees of iridotrabecular contact or peripheral anterior synechiae), IOP elevation (above 21 mmHg), glaucomatous optic neuropathy, and a corresponding visual field defect [15]. We excluded, from both groups, those (1) with other ocular diseases than cataract (and PACG for the cases), but we excluded intumescent cataract and mature cataract; (2) who had previously undergone laser treatment or ocular surgery; (3) who were experiencing an acute attack or had experienced an episode of acute attack within one month before surgery; (4) who had systemic diseases that could influence UA levels (e.g., kidney disease, diabetes, hyperuricemia, autoimmune disease, cardiac disease, and cancer); or (5) who were taking medications (e.g., intravenous mannitol, oral carbonic anhydrase inhibitors) that could influence UA levels. To further minimize the influence of food on UA levels, the participants from both groups were instructed not to consume any food that could influence UA levels (e.g., seafood, animal organs, beans, alcohol) for at least seven days prior to the scheduled surgery. These instructions were given when the patients made their surgery appointments in the outpatient department. When they were admitted for surgery, they were asked whether they had consumed any of these foods recently. Patients answering in the affirmative were not enrolled in the study. For analysis, the enrolled patients with PACG were subdivided into two groups: those with a history of acute primary angle closure (APAC) and those without a history of APAC. Based on the mean deviation (MD) of a visual field test, they were also subdivided according to the severity of glaucoma: mild (MD ≧ −6 dB), moderate (−12 dB ≦ MD < −6 dB), and severe (MD < −12 dB).

All the enrolled PACG patients underwent a standardized ocular examination, including assessment of vision acuity and refractive status, slit-lamp microscopy, fundoscopy, assessment of IOP (iCare-Pro, Icare Finland Oy, Finland), anterior chamber depth (ACD), central corneal thickness (CCT), axial length (AL), white-to-white distance (WTW), lens thickness (LT; LS 900 Biometer, Haag-Streit, Switzerland), and corneal endothelium cell density (ECD; SP-1P specular microscope, Topcon, Japan), gonioscopy, ultrasound biomicroscopy (UBM; MD-300L, MEDA, China), and visual field examination (Humphrey field analyzer with 30-2 grid and SITA Fast strategy). The controls underwent the same ophthalmic examinations, except for the UBM and visual field examinations.

Only one eye per patient/control was included. When both eyes fulfilled the inclusion criteria, the first eye scheduled for surgery was chosen.

### 2.2 Serum uric acid

Blood samples were collected via conventional venipuncture at the anterior elbow veins after a 10-h overnight fast. Serum UA levels were determined by enzymatic colorimetry using commercially available kits (Ningbo MedicalSystem Biotechnology Co., Ltd, Ningbo, China).

### 2.3 Aqueous humor uric acid

For PACG patients undergoing trabeculectomy or trabeculectomy combined with cataract surgery, aqueous humor (approximately 0.1 ml) was collected in a single sampling with a 26 G needle after the conjunctival and scleral flap were made, before any other incisional procedure was performed. No anti-metabolites were used before the aqueous humor sampling. For the controls, aqueous humor (approximately 0.1 ml) was collected in a single sampling with a 26 G needle at the beginning of the cataract surgery. A tank containing liquid nitrogen was present in each operation room, and all the collected aqueous humor samples were immediately stored in liquid nitrogen for further analysis.

Due to the limited volume of the aqueous humor sample, only a single analysis was performed on each sample. As described below, however, an identical technique and procedure was applied in both groups, thereby minimizing any bias that might result from systematic or random error in the analysis. The Human UA Enzyme-Linked ImmunoSorbent Assay kit (Mlbio Biotech, Shanghai, China) was applied to determine UA levels in aqueous humor according to the manufacturer's instructions. Briefly, the standard sample was used to produce a standard curve. Then 50 μl of the aqueous humor sample was diluted to 1:3 with sample diluent. Finally, the absorbance and the UA concentration was recorded using a microtiter plate reader (Infinite M200 PRO Multimode Microplate Reader, Tecan Trading AG, Switzerland). The enzyme-linked immunosorbent assay (ELISA) procedure was performed three times for each aqueous humor sample, and the average was calculated for the final analysis. The procedure has been described in detail elsewhere [16].

### 2.4 Statistical analyses

Characteristics of the subjects were described using means and standard deviations (SD) for normally distributed variables and using median and interquartile range (IQR) for skewed variables. The Kolmogorov–Smirnoff test was used to assess normality. A Student's t-test or Mann-Whitney test was used to compare groups, and a chi-square test was used to compare proportions. One-way ANOVA was applied to compare the aqueous humor UA levels across the three glaucoma-severity subgroups of PACG patients. To adjust for possibly confounding factors, we also assessed the association between PACG and aqueous humor UA levels using binary logistic regression models. In Model 1, we adjusted for age and gender. In Model 2, for age, gender, body mass index (BMI), diastolic blood pressure (DBP), systolic blood pressure (SBP), ACD, CCT, AL, WTW, LT, ECD, and serum UA level. The correlation between serum UA levels and aqueous humor UA levels in PACG patients and controls was assessed by Pearson correlation analysis. A two-tailed p-value of <0.05 was considered statistically significant.

## 3 Results

**Fig. 1:**
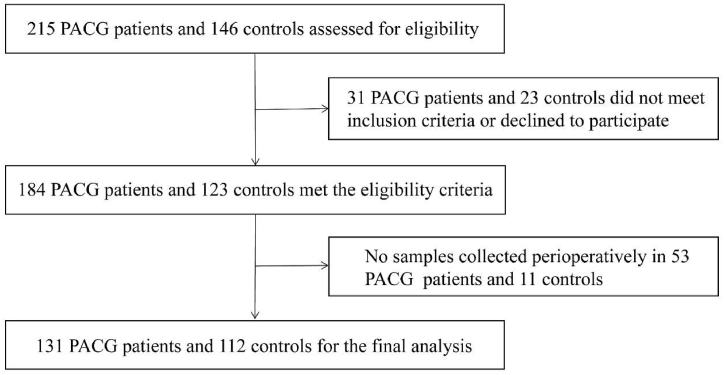
Flowchart showing inclusion and exclusion of primary angle closure glaucoma (PACG) patients and controls.

In all, 131 patients with PACG and 112 controls with cataract were enrolled (Fig. 1). Table 1 presents the demographics of the enrolled patients and controls. There were no statistical differences in age or gender between the patients with PACG and the controls.

**Table 1:** Baseline characteristics of the patients with primary angle closure glaucoma (PACG) and controls.

	PACG patients (n = 131)	Controls (n = 112)	p
Age (years)	69.3 ± 7.7	70.5 ± 8.1	0.21
Gender, female (n [%])	92 (70 %)	74 (66 %)	0.49
SBP (mmHg)	145 ± 18	143 ± 16	0.44
DBP (mmHg)	79.9 ± 10.6	78.2 ± 9.8	0.20
BMI (kg/m^2^)	24.7 ± 3.7	25.2 ± 3.7	0.27
AL (mm)	22.5 ± 0.8	23.3 ± 1.2	<0.001
CCT (μm)	538 ± 45	534 ± 34	0.48
ACD (mm)	1.71 ± 0.32	2.47 ± 0.40	<0.001
LT (mm)	4.99 ± 0.38	4.51 ± 0.42	<0.001
WTW (mm)	11.3 ± 0.5	11.4 ± 0.5	0.09
ECD (cells per mm^2^)	2507 ± 593	2716 ± 332	0.001
IOP (mmHg)	29.3 ± 7.5	14.3 ± 2.7	<0.001

SBP: systolic blood pressure; DBP: diastolic blood pressure; BMI: body mass index; AL: axial length; CCT: central corneal thickness; ACD: anterior chamber depth; LT: lens thickness; WTW: white-to-white distance; ECD: corneal endothelial cell density; IOP: intraocular pressure.

### 3.1 Serum uric acid levels

The serum UA level was 266 ± 69 μmol/L in the PACG group and 269 ± 73 μmol/L in the control group (Table 2; p = 0.71). The serum UA levels of all participants were within the reference range of UA in the healthy, elderly Chinese population (130–443 μmol/L for females and 179–461 μmol/L for males) [17]. In both groups, males had higher serum UA levels than females did (PACG patients: p = 0.002; controls: p < 0.001). The absence of a significant difference between PACG patients and controls persisted after stratification according to gender (male PACG patients versus male controls, p = 0.22; female PACG patients versus female controls, p = 0.48). The difference in serum UA level between PACG patients and controls also remained insignificant after adjusting for age and gender (Model 1; p = 0.86) and after adjusting for age, gender, SBP, DBP, BMI, AL, CCT, ACD, LT, WTW, ECD, and aqueous humor UA level (Model 2; p = 0.14).

**Table 2:** Serum and aqueous humor uric acid levels of the patients with primary angle closure glaucoma and the controls.

	Overall			PACG stratification			
	PACG patients (n = 131)	Controls (n = 112)	p	Mild (n = 15)	Moderate (n = 21)	Severe (n = 64)	p
Serum UA (μmol/L)							
Total	266 ± 69	269 ± 73	0.71	259 ± 76	263 ± 62	266 ± 76	0.95*
Male	295 ± 59	314 ± 72	0.22				
Female	254 ± 69	246 ± 63	0.48				
Aqueous humor UA (μmol/L)							
Total	35.4 ± 8.2	53.9 ± 18.6	<0.001	36.8 ± 16.0	33.9 ± 5.8	35.9 ± 8.8	0.57
Male	36.1 ± 8.3	53.7 ± 17.1	<0.001				
Female	35.0 ± 8.2	54.0 ± 19.4	<0.001				

UA: uric acid; PACG: primary angle closure glaucoma; *: p = 0.41 after adjusting for gender.

### 3.2 Aqueous humor uric acid levels

The aqueous humor UA level was 35.4 ± 8.2 μmol/L in the PACG group and 53.9 ± 18.6 μmol/L in the control group. This difference was statistically highly significant (p < 0.001). The difference in aqueous humor UA levels was also statistically significant after stratification according to gender (Table 2). Aqueous humor levels did not differ significantly between males and females in either the PACG patients or the controls (PACG patients: p = 0.49; controls: p = 0.95). There was no correlation between serum UA levels and aqueous humor UA levels in either the PACG patients (r = −0.01, p = 0.96) or the controls (r = 0.11, p = 0.25).

The relationship between PACG and aqueous humor UA level was further assessed with binary logistic regression models. The difference found in the univariable comparison remained significant after adjusting for age and gender (Model 1; odds ratio [OR]: 0.89, 95 % confidence interval [CI]: 0.86–0.91, p < 0.001), as well as after adjusting for age, gender, SBP, DBP, BMI, AL, CCT, ACD, LT, WTW, ECD, and serum UA level (Model 2; OR: 0.88, 95 % CI: 0.83–0.93, p < 0.001).

The aqueous humor UA level was 35.0 ± 6.3 μmol/L in PACG patients with a history of APAC (n = 61) and 35.7 ± 9.6 μmol/L in PACG patients without a history of APAC (n = 70). There was no statistically significant difference in aqueous humor UA level between these two groups (p = 0.66).

The PACG patients with reliable Humphrey visual field test results (n = 100) were subsequently divided into three subgroups according to disease severity, based on the MD: mild (n = 15), moderate (n = 21), and severe (n = 64). There were no differences in age (67.5 ± 5.5, 68.4 ± 7.1, and 68.3 ± 8.7 years in the mild, moderate, and severe groups, respectively; p = 0.93). The percentages of females were 93 % (14/15), 90 % (19/21), and 58 % (37/64) in the mild, moderate, and severe groups, respectively (p = 0.002). There was no statistical difference in serum UA and aqueous humor UA levels among the three groups (Table 2).

## 4 Discussion

In this study, we found a statistically significant difference in aqueous humor UA level, but not in serum UA level, between patients with PACG and controls. The difference in aqueous humor UA level remained statistically significant after adjustment for potential ocular and systemic confounding factors. To our best understanding, this is the first study to explore the aqueous humor UA levels of PACG patients.

Only two studies concerning the association between serum UA and PACG have been published, and both studies were conducted by the same research team, from China. In the retrospective case-control study conducted by this group [11], the serum UA level in patients with PACG (286 ± 82 μmol/L; n = 886) was significantly lower than it was in controls of similar age and gender distribution (295 ± 85 μmol/L; n = 994). The researchers also found a dose-response effect in this study; the severe PACG group presented the lowest serum UA level, followed by the moderate and mild group, thereby strengthening evidence that UA might be involved in the pathogenesis of PACG. In our study, no statistically significant differences in serum UA level were found, whether between PACG patients and control subjects, or between patient groups with different PACG severity. One possible reason is that our sample size is much smaller than that of Li et al. [11]. Importantly, although the main effect found by Li et al. [11] was statistically significant (due to the large sample size), it was small, that is, only approximately 0.1 SD (295−286 = 9 μmol/L, to be related to a standard deviation of 82 μmol/L in the patients and 85 μmol/L in the controls). A seemingly larger effect was found in the prospective cohort study in which Li et al. investigated the association between baseline serum UA levels and the progression of recently diagnosed PACG [12]. The researchers found that non-progressing subjects had higher baseline UA values (315 ± 71 μmol/L; n = 32) than progressing subjects did (256 ± 71 μmol/L; n = 32); the groups were balanced in gender and age. Although the design of this study precludes a direct comparison to our data, a tentative interpretation of the data from both studies of Li et al. [11,12] and our data could be that serum UA is not associated with the onset of PACG (anatomical factors are presumably more important in this regard), but that a high serum UA level is beneficial to slowing down disease progression in existing PACG. Of the various roles that UA may play in glaucoma, a pro-oxidant and an antioxidant role, the second role seems to dominate in this regard.

**Fig. 2:**
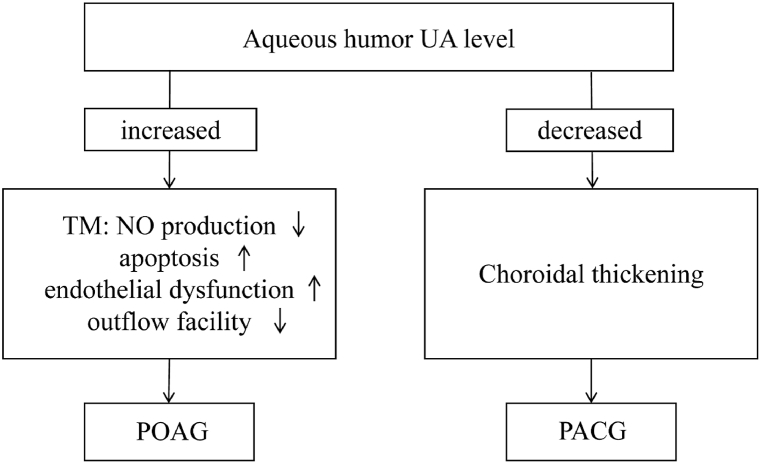
Possible mechanisms underlying aqueous humor uric acid level and glaucoma (UA: uric acid; TM: trabecular meshwork; POAG: primary open angle glaucoma; PACG: primary angle closure glaucoma).

To date, the literature has contained only limited data regarding aqueous humor UA levels in glaucoma. In one study, using a rabbit model with congenital glaucoma, significantly lower aqueous humor UA levels were found in the glaucoma rabbits than in age-matched controls [18]. In another study, POAG patients showed increased aqueous humor UA levels compared with controls [7]. We found statistically significantly lower aqueous humor UA levels in PACG patients than in controls, and this difference remained statistically significant after adjustment for potential ocular and systemic confounding factors. The inconsistency between the latter study and our data could be related to differences in glaucoma type (POAG versus PACG). The TM is the main pathway for aqueous humor outflow, and its impairment is responsible for the elevated IOP found in POAG [19]. High UA levels in the aqueous humor of POAG patients can reduce the influence of ascorbate on the molecular property of glycosaminoglycans in the TM, thus impairing its outflow facility and leading to increased IOP [20]. An elevated UA level can also induce endothelial dysfunction of TM cells by decreasing NO production and mediating apoptosis, thus facilitating the development of POAG [7]. The elevated aqueous humor UA level in POAG patients could also be secondary. In other words, it could be the result of intraocular compensatory mechanisms to counteract oxidative damage [21], which are considered to contribute to the pathogenesis of POAG [22]. The role of local UA in PACG may be different from its role in POAG. A crowded anterior segment and narrow anterior chamber angle are the anatomic characteristics leading to PACG. In a recent study, an elevated UA level was associated with impaired microcirculation in retina and choroid [23]. If the opposite were also to be the case, then a decrease in aqueous humor UA level would enhance retinal and choroidal microcirculation. The resulting increase in choroidal thickness/volume could subsequently contribute to the occurrence of PACG [2]. The possible underlying mechanisms are illustrated in Fig. 2.

We acknowledge several limitations of our study. First, our controls were patients with cataract rather than subjects with healthy eyes. It was primarily for logistic and ethical reasons that we included controls that required surgery. Cataract has been associated with elevated aqueous UA levels, especially in subcapsular posterior cataract [24]. At the same time, however, the choice to have patients with cataract as controls could be considered a strength as well, given that lenticular changes also contribute to PACG. As such, patients with cataract might form a better control group than subjects with healthy eyes to adjust for any potential influence of UA on cataract formation. Second, our patient group consisted of many more patients with advanced PACG (n = 64) than with mild (n = 15) or moderate (n = 21) disease. This might be the reason why we failed to find any dose-response relationship. Another reason could be that we had reliable visual fields, needed to stage the patients, only in a subgroup of 100 patients. A well-designed study with a larger sample size that properly represents the entire disease severity spectrum is needed to further explore the dose-response relationship of UA. Third, UA levels might be influenced by IOP [25]. It is unlikely that this effect had any substantial influence on our results. This effect has been reported only for women under the age of 50. All our participants were more than 50 years old, and we did not observe any gender-specific effects. Moreover, our patients did not undergo surgery during an acute attack, but later, after normalization of the IOP. Fourth, our study utilized ELISA analysis for each sample, and the aqueous humor UA level in our controls (cataract patients) was much lower than that determined by chromatography [21] or automatic analyzers [24,26]. However, our result (53.9 ± 18.6 μmol/L) was comparable to that determined by enzymatic-colorimetry (62.6 ± 17.4 μmol/L) [7], a technique akin to ELISA. This discrepancy in results might be attributed to variations in the assessment methods employed. Importantly, we employed a uniform technique and procedure in both groups during our study, thereby minimizing the potential bias arising from systematic or random error in the analysis.

One strength of our study is that, to our best understanding, it is the first to investigate aqueous humor UA levels in PACG patients. Uric acid is present in several body fluids, including urine, plasma, serum, and aqueous humor. It is now regarded as an important biomarker of various diseases, including chronic kidney disease, cardiovascular diseases, metabolic syndrome, neurological diseases, and psychiatric disorders [[27], [28], [29]]. Our findings indicate that aqueous humor UA may serve as a biomarker of PACG. Given the difficulty of measuring aqueous humor UA levels in clinical practice, however, aqueous humor UA is unlikely to be assessed in the management of glaucoma. In addition, aqueous humor UA levels cannot be predicted from serum UA levels. In our study, aqueous humor UA levels were only weakly correlated with serum UA levels and, on average, they were much lower than serum UA levels. Moreover, a gender difference was present for serum but not for aqueous humor UA levels. All these findings indicate that serum and aqueous humor UA levels are largely independent of each other. Although aqueous humor UA is difficult to assess in clinical practice, studying aqueous humor UA could advance the understanding concerning the pathophysiology of glaucoma, and may thus eventually improve glaucoma care. For example, UA might be considered a therapy target for glaucoma and modulation of UA levels might help to protect the retinal ganglion cells. In addition to clinical studies, basic research (such as cell experiments or animal models) is needed for this. Finally, information regarding choroidal thickness was not available in this study. A future study exploring the association between UA levels and choroidal thickness is needed to confirm or falsify our hypothesis and provide clearer insight into the underlying mechanisms.

## 5 Conclusion

Aqueous humor UA levels differ between patients with PACG and controls, but serum UA levels do not. This indicates that local UA plays a role in the pathogenesis of PACG, but systemic UA does not. Aqueous humor UA might be considered a biomarker and a potential therapy target of PACG. A study involving a more uniform distribution of glaucoma severity across the cases could identify the existence of a more subtle dose-response effect of aqueous humor UA in patients with PACG, thus advancing understanding concerning the role of UA in PACG.

## Ethics approval and consent to participate

This research followed the tenets of the Declaration of Helsinki. Written informed consent was obtained from all the subjects, following explanation of the nature and possible consequences of the study. This study was approved by the ethics committee of Tianjin Medical University Eye Hospital (2018KY-02).

## Consent for publication

Not applicable.

## Availability of data and materials

The datasets used and/or analyzed during the current study are available from the corresponding author on reasonable request.

## Funding

The study received financial support from the Open Project of 10.13039/501100015340Tianjin Key Laboratory of Retinal Functions and Diseases (2020tjswmq003); the Youth Special Fund of Clinical Research of Tianjin Medical University Eye Hospital (2020QN02); the Tianjin Key Medical Discipline (Specialty) Construction Project (TJYXZDXK-037A), and the University of Groningen Abel Tasman Talent Program (University Medical Center Groningen/Tianjin Medical University). These funding organizations had no role in the design or conduct of this research.

## CRediT authorship contribution statement

**Wei Liu:** Writing – review & editing, Writing – original draft, Methodology, Funding acquisition, Formal analysis, Conceptualization. **Ruru Guo:** Formal analysis, Data curation, Funding acquisition, Writing – original draft, Writing – review & editing. **Fei Gao:** Writing – review & editing, Formal analysis, Methodology. **Dandan Huang:** Writing – review & editing, Formal analysis. **Xinyi Zhang:** Writing – review & editing, Data curation. **Jian Ji:** Writing – review & editing, Supervision, Formal analysis, Conceptualization. **Nomdo M. Jansonius:** Writing – review & editing, Supervision, Methodology, Formal analysis, Conceptualization.

## Declaration of competing interest

The authors declare that they have no known competing financial interests or personal relationships that could have appeared to influence the work reported in this paper.

## List of abbreviations

UA: uric acid
PACG: primary angle closure glaucoma
POAG: Primary open angle glaucomaI
IOP: intraocular pressure
TM: trabecular meshwork
MD: mean deviation
CCT: central corneal thickness
ACD: anterior chamber depth
AL: axial length
LT: lens thickness
WTW: white-to-white distance
CED: corneal endothelium cell density
UBM: ultrasound biomicroscopy
ELISA: Enzyme-Linked ImmunoSorbent Assay
APAC: acute primary angle closure
SBP: systolic blood pressure
DBP: diastolic blood pressure
BMI: body mass index
